# Inversion symmetry of DNA k-mer counts: validity and deviations

**DOI:** 10.1186/s12864-016-3012-8

**Published:** 2016-08-31

**Authors:** Sagi Shporer, Benny Chor, Saharon Rosset, David Horn

**Affiliations:** 1 Blavatnik School of Computer Science, Tel Aviv University, Tel Aviv, 69978 Israel; 2 Sackler School of Mathematical Sciences, Tel Aviv University, Tel Aviv, 69978 Israel; 3 Sackler School of Physics and Astronomy, Tel Aviv University, Tel Aviv, 69978 Israel

**Keywords:** Generalized Chargaff rules, Chromosome k-mer distributions, Inversion symmetry

## Abstract

**Background:**

The generalization of the second Chargaff rule states that counts of any string of nucleotides of length k on a single chromosomal strand equal the counts of its inverse (reverse-complement) k-mer. This Inversion Symmetry (IS) holds for many species, both eukaryotes and prokaryotes, for ranges of k which may vary from 7 to 10 as chromosomal lengths vary from 2Mbp to 200 Mbp. The existence of IS has been demonstrated in the literature, and other pair-wise candidate symmetries (e.g. reverse or complement) have been ruled out.

**Results:**

Studying IS in the human genome, we find that IS holds up to k = 10. It holds for complete chromosomes, also after applying the low complexity mask. We introduce a numerical IS criterion, and define the k-limit, KL, as the highest k for which this criterion is valid. We demonstrate that chromosomes of different species, as well as different human chromosomal sections, follow a universal logarithmic dependence of KL ~ 0.7 ln(L), where L is the length of the chromosome.

We introduce a statistical IS-Poisson model that allows us to apply confidence measures to our numerical findings. We find good agreement for large k, where the variance of the Poisson distribution determines the outcome of the analysis. This model predicts the observed logarithmic increase of KL with length. The model allows us to conclude that for low k, e.g. k = 1 where IS becomes the 2^nd^ Chargaff rule, IS violation, although extremely small, is significant. Studying this violation we come up with an unexpected observation for human chromosomes, finding a meaningful correlation with the excess of genes on particular strands.

**Conclusions:**

Our IS-Poisson model agrees well with genomic data, and accounts for the universal behavior of k-limits. For low k we point out minute, yet significant, deviations from the model, including excess of counts of nucleotides T *vs* A and G *vs* C on positive strands of human chromosomes. Interestingly, this correlates with a significant (but small) excess of genes on the same positive strands.

**Electronic supplementary material:**

The online version of this article (doi:10.1186/s12864-016-3012-8) contains supplementary material, which is available to authorized users.

## Background

Erwin Chargaff has made, in 1950, the important observation that the numbers of nucleotides in DNA satisfy #A = #T and #G = #C [1, 2]. This statement, made on the basis of experimental observations with fairly large errors, played a crucial role in realizing that DNA has an underlying base-pair grouping, as subsequently proposed by Crick and Watson [3] in their double-helix structure.

The second Chargaff rule [4] states that the same sets of identities of nucleotide pairs hold for each long enough *single* DNA strand. This rule has been tested [5] for genome assemblies of many species, and found to be globally valid for eukaryotic chromosomes, as well as for bacterial and archaeal chromosomes. It fails for mitochondria, plasmids, single-stranded DNA viruses and RNA viruses.

The validity of the second Chargaff rule was unexpected. Obviously it should be regarded as a global rule, i.e. applicable to large sections of chromosomes. Nonetheless, not being derived from a compelling principle, such as the one underlying the first rule, it remains a mystery. This is even more so, when one studies extended versions of Chargaff’s second rule. Indeed, Albrecht-Buehler [6] observed that for triplet oligonucleotides, or 3-mers, it remains true that their chromosome-wide frequencies are almost equal to those of their reverse-complement 3-mers. Prabhu [7] has shown that this symmetry holds up to 5-mers in various species. This has been reviewed by Baldi and Brunak [8] who have argued that such symmetry rules have to be incorporated in Markov models of genomic sequences.

We refer to the symmetry between counts of k-mers and their reverse complements as

***Inversion Symmetry (IS)****: the counts of a k-mer of nucleotides on a chromosomal strand are almost equal to those of its inverse (reverse-complement) string.*

Note that this implies that the number of times a string of nucleotides of length *k* is observed on a strand, when read from 5’ to 3’, is almost equal to the number of times it is observed on the other strand when the latter is read from its 5’ end to 3’ end.

Recent analyses of inversion symmetry include the following: Qi and Cuttichia [9] who have shown that inversion symmetry exists while reverse symmetry fails, i.e. k-mers and their reverses do not appear with equal rates; Baisnee, Hampson and Baldi [10], who introduced a measure S1 to analyze inversion symmetry in a systematic fashion; Kong et al. [11], who established the validity of IS on 786 chromosomes of many species and showed that reverse or complement symmetry do not hold, and argued that IS may be due to segmental or whole-genome inverse duplications; Wang et al. [12] who argued that values of k for which k-mer IS is valid increase with organismal complexity; and Afreixo et al. [13] who applied various criteria to demonstrate the statistical significance of IS up to k = 10. Studies of symmetries related to IS appear in [14, 15].

We introduce an IS measure which is different from S1 of [10], albeit the numerical results of both measures are correlated (see section 4 in Methods). Our measure is based on the ratio between differences of counts of inverse k-mer pairs and their sum. We propose the criterion that if the average of this normalized measure (over all strings of length k) is less than about 0.1, IS will be regarded as a valid approximate symmetry. The average is taken over all M_k_ ≤ 4^k^ strings of length k which exist at least once on the chromosome. The value of k, for which the IS measure is closest to 0.1, is defined as the k-limit (KL). This turns out to be KL = 10 on long human chromosomes (see Additional file 1), and KL = 7 or KL = 8 for bacteria.

Using this measure, one can readily demonstrate the existence of inversion symmetry, and the absence of analog symmetries between reverse pairs or complement pairs, as well as compare between different species. We will show that the k-limit of inversion symmetry, KL, is logarithmically dependent on the length L of the chromosome, or of a chromosomal section on which it is measured. Moreover, this dependence is universal, i.e. it is valid for most species.

To analyze all these observations on a rigorous statistical basis, we introduce a Poisson model for the random occurrence of counts, regarding *N*(S) as a stochastic variable for any string S of length k. We define *X*(S,S^*^) = |*N*(S)-*N*(S^*^)|/(*N*(S) + *N*(S^*^)), which is a stochastic variable having positive values 0 ≤ X ≤ 1. In general S^*^ is some permutation of S over the set of all strings of length k. When S^*^ ≡ S^inv^, i.e. where S^*^ is the inverse of S, IS implies that X < <1. For strict IS, X = 0. In practice we may observe small deviations when checking for its realization on a chromosome. The important question we address is whether these deviations mean that the IS rule is not valid, or that the data are consistent with IS yet the observed values of X reflect statistical fluctuations.

To answer this question we introduce the stochastic variable *Z*(S,S^*^) = (*N*(S)-*N*(S^*^))/(*N*(S) + *N*(S^*^))^½^. The symmetry assumption means that *N*(S) = *N*(S*), i.e. these two stochastic variables have the same distribution. If we further assume that *N*(S) ~ *Poisson* then *Z* should be approximately distributed as a standard normal (see Methods).

We will demonstrate on genomic data that for inversion symmetry we empirically observe that *Z* ~ *Standard Normal*, but for other pairings of S and S* (e.g. reverse or complement) it is not. Continuing with the analysis of IS we show that

- For small k, E_k_[X] is extremely small, yet E_k_[|Z|] = E_k_[X/σ_X_] > 2, where σ_X_ is the (theoretically estimated) standard deviation of *X*(S,S^inv^). Therefore we conclude that there exists a systematic small breaking of IS, observed for k < 4 on human chromosomes.
- For large k (k > 5 on human chromosomes) E_k_[|Z|] = E_k_[X/σ_X_] < 1 hence, due to the large variance, one may state that the observed X values are consistent with IS. Moreover, the data are consistent with *Z ~ Standard Normal* for large k.
- The empirical values of X(S,S^inv^) for large k are of the order of magnitude of (N(S) + N(S^inv^))^-½^.
- The logarithmic variation of the k-limit, KL, as function of chromosomal length L, is correctly predicted by our IS-Poisson model.

We use the italicized notation *N, X, Z,* for the stochastic variables of our model, and employ N, X, Z for their empirical counts on chromosomes. KL is defined as the value of k for which E_k_[X] is closest to 0.1.

## Results

### Inversion symmetry (Generalized 2^nd^ Chargaff Rule)

Let S and S^*^ be two strings of nucleotides of same length k, i.e. two k-mers. Suppose they appear N(S) and N(S^*^) times respectively on a particular chromosome. We denote by X(S,S^*^) the normalized difference X(S,S^*^) = |N(S)-N(S^*^)|/(N(S) + N(S^*^)) where S is one of the M_k_ different k-mers over the 4 nucleotides, which are being counted on the chromosome at least once, i.e. N(S) > 0 and/or N(S^*^) > 0. If both N(S) = N(S^*^) = 0, X(S,S^*^) is defined to be 0. In general 0 ≤ X(S,S^*^) ≤ 1.

We use E_k_[X] = ∑_S_ X(S,S^*^)/M_k_, where M_k_ is the number of different k-mers encountered empirically, as a measure to demonstrate and quantify the studied symmetry. For low and moderate k, we find that M_k_ = 4^k^, but for large k-values, such as k > 10 in the human genome, many of the k-mers may not be realized empirically, leading to lower M_k_. In the following we will look at values of X(S,S^*^) over various possible choices of string pairs S and S^*^, and demonstrate that for inverse pairs they are distributed differently than for other types of k-mer pairs.

**Fig. 1:**
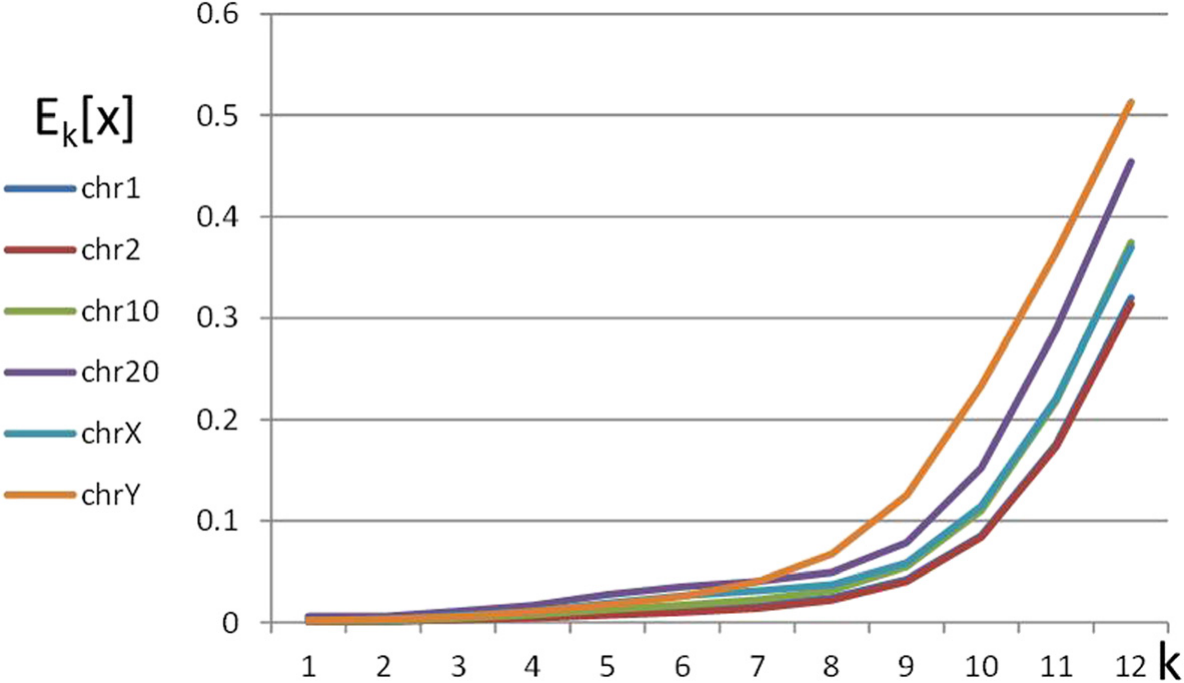
Averages of normalized differencess between occurrences of k-mers and their inverses (reverse-complements), E_k_[X], for different chromosomes of the HG38 human assembly, plotted *vs* k

Let us start by computing E_k_[X] for inverse pairs (i.e. S and S^*^ ≡ S^inv^ are reverse-complements of each other) for different k, on various chromosomes of the human genome assembly HG38. Data were downloaded from the UCSC genome browser http://genome.uscs.edu. The values of E_k_[X] for several human chromosomes are displayed as function of k in Fig. 1. Inversion Symmetry (IS) is seen to hold quite well for k-mers with large k-values for all the displayed chromosomes. Chr Y, which is the shortest among the 24 chromosomes, has the least inversion symmetry. IS holds also for all other chromosomes (Additional file 1). It fails for the mitochondrial chromosome, which is a well-known exception to the 2^nd^ Chargaff rule.

Repetitive structures are well-known to constitute major fractions of eukaryotic chromosomes, hence one may wonder to what extent they are responsible for the observed inversion symmetry. To resolve this issue, we employed the same operations on the masked output of the UCSC genome browser, after filtering chromosomes for interspersed repeats and low complexity sequences. The results keep displaying the same behavior, with negligible differences for high values of k. Even chrY, which is well known for containing numerous repeats, with only 36 % of it surviving the masking filter, keeps showing the same qualitative behavior as in Fig. 1. In Additional file 1 we provide a list of the highest k-values for which E_k_[X] ≈ 0.1, both before and after masking (which removes repetitive and low-complexity stretches of the chromosome). We define the k-limit (KL) of IS, as the value of k for which E_k_[X] is closest to 0.1. The observed reduction in KL from 10 to 9 for the largest chromosomes, is due to the fact that masking shortens the effective chromosome length. The dependence of KL on length is an issue to which we will return below.

We have performed the same analysis on the older genome assembly HG18, leading to very similar results (see Additional file 2). We find similar IS results for mouse, frog, fly, worm, and yeast. Moreover, we find that inversion symmetry holds also for bacteria, but it is valid for a lower range of k-mers, only up to KL = 6 or 7.

### Outstanding features of inverse k-mer pairs

In order to demonstrate how Inversion Symmetry, observed for frequencies of inverse pairs, differs from other natural pairings, we compare different choices of pairings of k-mers,

- Inverse pairs (e.g. CGA *vs* TCG)
- Random pairs
- Reverse pairs (e.g. CGA *vs* AGC)
- Complement pairs (e.g. CGA *vs* GCT)

We have evaluated histograms of X(S,S^*^) = |N(S)-N(S^*^)|/(N(S) + N(S^*^)) for all pairings, and computed their averages E_k_[X] = 4^-k^∑_S_ X(S,S^*^) for different k. Calculations were performed both for human chromosomes as well as for many other species.

**Fig. 2:**
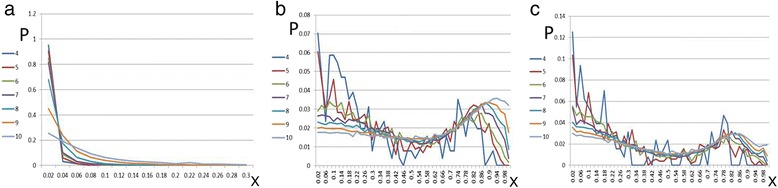
HG38 chr1: Histogram (probability distribution in bins of Δx = 0.02) of relative occurrences of k-mer pairs *vs* x for different values of k (4 to 10). **a** inverse pairs; plotted range is x < 0.3, above which the histogram values are negligibly small. **b** random pairs for full x range; **c** Reverse pairs for full x range

Figure 2a depicts the distributions of X values for inverse pairs on human chr 1 of HG38, evaluated for k = 4 to 10. These distributions are very narrow, leading to very low E_k_[X] values, consistent with the results displayed in Fig. 1. As k increases they widen, leading to increasing E_k_[X] values, which will be discussed below and are quoted in Table 1. In Fig. 2b and c we plot the corresponding distributions for the cases of random pairs (b), where for each S a random choice of S^*^ is being made, without repetition, and reverse pairs (c) on chr 1. Distributions of complement pairs (d) are identical to those of reverse pairs and are therefore not displayed as an additional figure. Note that the distributions in 2b and 2c are completely different from 2a: they possess a rugged wavy behavior, stretching over the whole range of 0 < X < 1. Since k-mer distributions on the human genome are known to be different for strings containg CG dimers [16], we studied the same problems removing all such k-mers. It turns out that, for the resulting k-mer strings, the second peak in (b) and (c) disappears. But, even then, cases b and c continue to be very different from case a, displaying long tail distributions. Such characteristic differences occur also for all other species that we have tested, and also for masked chromosomes in human.

**Table 1:** comparisons of averages E_k_[X] of μ_ka_ = inverse pairs, μ_kb_ = random pairs, and μ_kc_ = reverse pairs, for chr1 of HG38

k	μ_ka_	μ_kb_	μ_kc_
1	0.0009	0.083	0
2	0.0008	0.20	0.15
3	0.0031	0.26	0.21
4	0.0055	0.33	0.27
5	0.0090	0.40	0.32
6	0.013	0.44	0.36
7	0.017	0.49	0.40
8	0.025	0.52	0.43
9	0.043	0.55	0.46
10	0.085	0.57	0.49
11	0.18	0.60	0.53
12	0.32	0.67	0.60

We can further use these distributions to establish that a symmetry relation holds only for inverse pairs, leading to very low E_k_[X] values, and not for any other pairing. Table 1 lists the values of μ_k_ = E_k_[X] for the three cases a, b and c, making it quite evident that IS holds and other symmetries do not. We will not dwell on it further, since Kong et al. [11] have already established the validity of IS (albeit using different measures) on 786 chromosomes of various species, and showed that reverse or complement symmetries do not hold.

### Statistical analysis of inversion symmetry

In the Methods section we point out that, for large enough counts, if the counts N(S) and N(S*) are drawn from the same distribution, then the variable Y = (N(S) - N(S*))/(N(S) + N(S*)) should have an approximately Gaussian distribution with mean 0 and standard deviation σ_G._ Moreover, the distribution of X = |Y| will have an expectation value E_k_[X] = 0.8 σ_G_ and standard deviation σ_X_ =0.6 σ_G_. If the counts N(S) are drawn from a Poisson model, we expect for each pair to find σ_G_ = (N(S) + N(S*))^-½^. Hence Z = (N(S) - N(S*))/(N(S) + N(S*))^½^ should follow a standard normal distribution, i.e. a Gaussian with mean = 0 and variance = 1. Hence the IS-Poisson model predicts E_k_(|Z|) = 0.8 and σ_k_(|Z|) = 0.6, when rounded up to first decimal point.

We have tested this model by evaluating results for inverse pairs of k-mers on chr1 of HG38. The results are displayed in Table 2.

**Table 2:** Results of the evaluation of averages and variances over k-mers of X and Z distributions on human chr 1. Large k-values approach the results E_k_(|Z|) = 0.8 and σ_k_(|Z|) = 0.6 expected from standard normal Z distributions

k	E_k_[X]	E_k_[|Z|] = E_k_[X/σ_X_]	σ_k_[|Z|]
1	.0004	4.56	3.7
2	.0006	3.26	2.4
3	.00075	1.98	1.58
4	.00125	1.34	1.12
5	.002	1.07	.86
6	.004	.93	.75
7	.0085	0.89	.72
8	.018	0.866	.72
9	.038	0.843	.69
10	0.083	0.825	.67

For low k-values, where E_k_[|Z|] = E_k_[X/σ_X_] ≥ 2, one may say that a mathematical hypothesis of strict IS is invalid, since the peak of the Z-distribution lies outside the allowed confidence interval. On the other hand, clearly for all k < 4, E_k_[X] < <0.01. Although the violation of IS is very small numerically, it is still statistically significant.

For large k > 5 we see that the data tend toward the prediction of our IS-Poisson model, approaching the limit of E_k_(|Z|) ± σ_k_(|Z|) = 0.8 ± 0.6. This means that, due to the large variance, arising from relatively small values of N(S) + N(S^inv^), the mathematical IS hypothesis cannot be refuted. It also means that variance plays a dominant role leading to the observed values of X(S,S^inv^) which are of the order of magnitude of (N(S) + N(S^inv^))^-½^. This implies that we should be able to deduce the behavior of the k-limit, which is indeed the case as will be shown below.

**Fig. 3:**
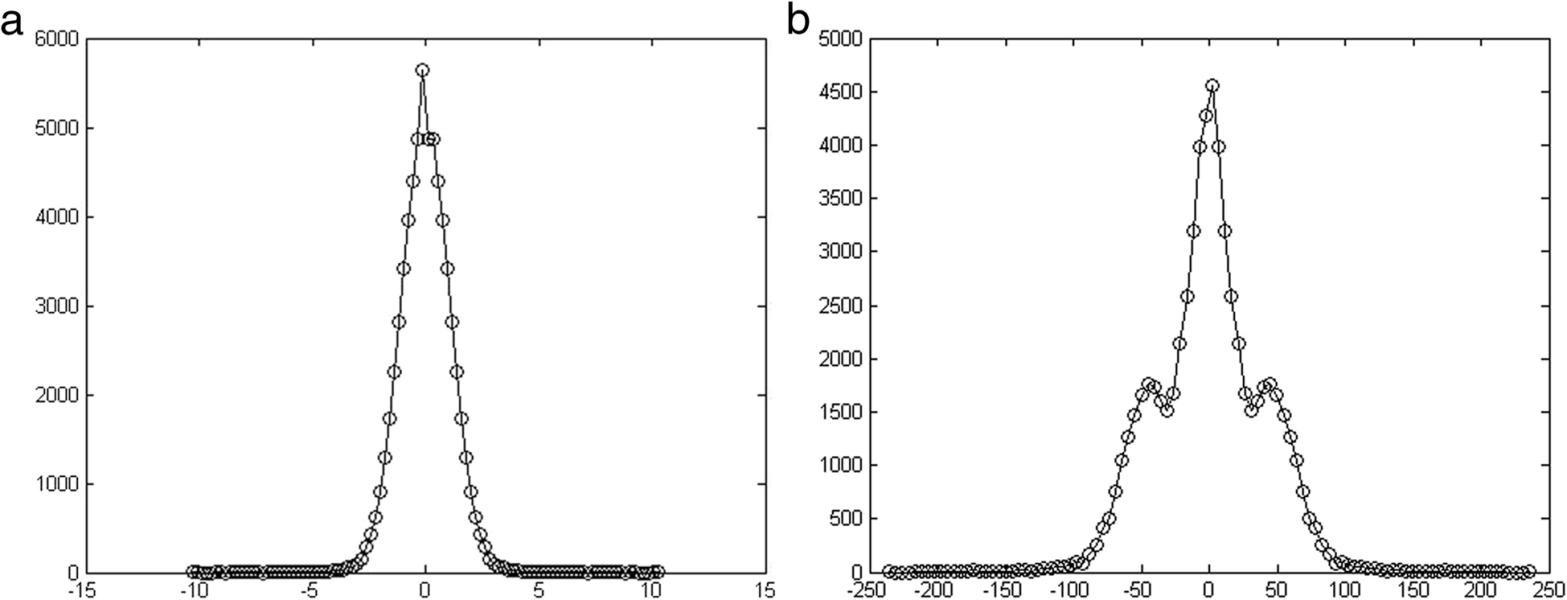
**a** Z-Distrbution for inverse kmer pairs of k = 8 shows high consistency with the expected standard normal distribution. **b** Z-Distribution of reverse pairs of k = 8 displays a completely different behavior from inverse pairs, having variance = 1600

To get a visual confirmation of the Gaussian nature of the Z-distribution we plot in Fig. 3a the results for k = 8 on human chr 1. The ensemble of Z-values contributed by all k-mers makes up the Gaussian distribution which is displayed here. The variance calculated from these data is 1.27, quite close to the value 1 expected from a standard normal distibution. For comparison, we display in Fig. 3b the analogous distributon of reverse pairs, which has variance of 1600. Note the different scales and shapes, which reflect the large difference between inverse and reverse distributions. The complement-pair distribution (not shown here) is essentially identical to the reverse one.

Considering the peak at Z = 0 displayed in Fig. 3b, it is important to note that there exists a subset of k-mers which obey S = S^rev^, i.e. they are palindroms. They will contribute to the peak at Z = 0, with small variations of palindroms contributing to the region around this peak. Nonetheless, their numbers are small compared to all 8-mers: only 3224 out of 65536 8-mers lie within |Z| < 1.65, which is where 90 % of a standard normal distribution are expected to reside. Hence the variance of the reverse-pair Z-ditribution is very large.

### Inversion symmetry for chromosomal sections

We next test to what degree IS is valid within various sections of human chromosomes. In Additional file 3, we display a characteristic distribution of inverse pairs drawn from a section of length 10Mbp, and in Additional file 4 we show an analogous distribution for length of 1Mbp. The IS quality, as determined by our convention, deteriorates leading to lower k-limits as the length of the section decreases, but it remains valid. The distributions in Additional file 4 are evidently noisier than their analogs in Additional file 3; however they are much narrower than those of the reverse and random pairs (not shown here).

To study systematically different sections of chromosomes, we evaluate the E_k_[X] values of inverse, random and reverse pairs, on non-overlapping windows of given lengths L. In general, inverse-pairs lead to smaller E_k_[X] than the other pairing choices. To determine the k-limit we impose the condition E_k_[X] <0.1 on the average over all chromosomal sections. The example displayed in Additional file 5 is of chr1, which is being tested with windows of length L = 5Kbp for inverse-pairs of k = 2. Although the average value is 0.07, obeying our criterion for IS validity, it is quite obvious that on many 5 K windows the values are higher. The value KL = 2 is chosen as the k-limit of IS validity in this case. Reducing the section length further down to L = 1Kbp, in Additional file 6, we find that IS fails even at order k = 1, i.e. the second Chargaff rule does not hold for such short sectors.

**Fig. 4:**
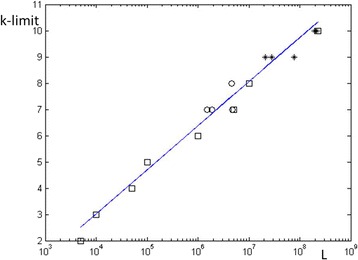
k-limits *vs* chromosomal length, based on Table 3. The figure displays a universal logarithmic behavior. Boxes are human data, stars denote examples of other eukaryotes, and circles represent examples of prokaryotes. The shown linear regression of this set of data has a slope of 0.73*ln(length), which agrees with our theoretical expectation

Similar evaluations for different chromosomes, on both HG18 and HG38 assemblies, lead in a consistent manner to the k-limits of “human sections” displayed in Table 3, where they are compared with results obtained for various other species, both eukaryotes and prokaryotes. They all follow a logarithmic increase of KL as function of the length of the chromosomal section, as is quite evident from their display in Fig. 4.

**Table 3:** k-limits for human data as well as other eukaryotes and prokaryotes

Species	Length	KL
HG38 chr1	230 M	10
HG18 chr1	225 M	10
Chimpanzee chr1	217 M	10
Mouse chr1	192 M	10
HG18 chrX	151 M	9
Zebrafish chr7	77 M	9
D. melanogaster chr3R	28 M	9
C. elegans chrV	21 M	9
HG18 chrY	26 M	8
Human section 10 M	10 M	8
E. coli K12	4.6 M	8
B. subtilis	4.2 M	8
Human section 5 M	5 M	7
M. avium paratubercolosis	4.8 M	7
P. furyosus	1.91 M	7
T. maritima	1.86 M	7
S. cerevisiae chr IV	1.53 M	7
Human section 1 M	1 M	6
Human section 100 K	100 K	5
Human section 50 K	50 K	4
Human section 10 K	10 K	3
Human section 5 K	5 K	2

The logarithmic increase is modelled well by our IS-Poisson model. To prove it let us define N(S) = f(S) L /4^k^, and let us assume that E[|Z|] reaches its asymptotic value 0.8. We may then rewrite

$$ \begin{array}{l}\mathrm{E}\left[\mathrm{X}\right]={4}^{{\textstyle \hbox{-}}\mathrm{k}}{\displaystyle {\sum}_{\mathrm{S}}}\left|\mathrm{N}\left(\mathrm{S}\right){\textstyle \hbox{-}}\mathrm{N}\left({\mathrm{S}}^{\mathrm{inv}}\right)\right|\Big(\mathrm{f}\left(\mathrm{S}\right)+\mathrm{f}{\left({\mathrm{S}}^{\mathrm{inv}}\right)}^{{\textstyle \hbox{-}}\raisebox{1ex}{$1$}\!\left/ \!\raisebox{-1ex}{$2$}\right.}{\left({4}^{\mathrm{k}}/\mathrm{L}\right)}^{\raisebox{1ex}{$1$}\!\left/ \!\raisebox{-1ex}{$2$}\right.}/\Big(\mathrm{N}\left(\mathrm{S}\right)+\mathrm{N}{\left({\mathrm{S}}^{\mathrm{inv}}\right)}^{\raisebox{1ex}{$1$}\!\left/ \!\raisebox{-1ex}{$2$}\right.}\\ {}\approx \left\{{4}^{{\textstyle \hbox{-}}\mathrm{k}}{\displaystyle {\sum}_{\mathrm{S}}}\right|\mathrm{N}\left(\mathrm{S}\right){\textstyle \hbox{-}}\mathrm{N}\left({\mathrm{S}}^{\mathrm{inv}}\right)\Big|{\left(\mathrm{f}\left(\mathrm{S}\right)\right)}^{{\textstyle \hbox{-}}\raisebox{1ex}{$1$}\!\left/ \!\raisebox{-1ex}{$2$}\right.}/\Big(\mathrm{N}\left(\mathrm{S}\right)+\mathrm{N}{\left({\mathrm{S}}^{\mathrm{inv}}\right)}^{\raisebox{1ex}{$1$}\!\left/ \!\raisebox{-1ex}{$2$}\right.}\Big\}{\left({4}^{\mathrm{k}}/2\mathrm{L}\right)}^{\raisebox{1ex}{$1$}\!\left/ \!\raisebox{-1ex}{$2$}\right.}\end{array} $$

The expression in {} is the expectation value of (f(S))^-½^ within the |Z|-distribution. Let us denote it by 0.8c_k_ since E_k_(|Z|) = 0.8. It follows that E_k_[X] < 0.1 means that 0.8 c_k_ (4^k^/2L)^½^ < 0.1. If c_k_ is a slowly varying function of k then k < lnL/ln4 + const = 0.72lnL + const.

This result is evidently borne out by the experimental fit k < 0.73 ln(L) + const. in Fig. 4. Furthermore, studying human chr1 we find that the experimental averages of (f(S))^-½^ (without weighting by |Z|) obtain the values 1.24, 1.45, 1.47 for k = 4, 6, 8 respectively. This verifies that c_k_ is indeed a slowly varying function.

An early observation of inversion symmetry measures increasing logarithmically with sequence size has been made by [10] for various DNA and RNA sequences (see Fig. 1 in [10]).

### Modeling inversion symmetry

If IS holds exactly for some k = k_0_, it will hold also for all k < k_0_, since the latter are substrings of the former and, therefore, all the frequencies of the k inverse-pair substrings will be matched (since the frequencies of their k_0_ hosts are being matched). In Methods we show that this statement is also true when IS is approximately true, i.e. when E_k_ [X] < <1. In practice we find it to hold when we apply our criterion E_k_ [X] ≤0.1 (see Table 4). One may wonder to what extent the opposite may hold within, e.g., low order Markov models: will a Markov model, constructed such that it satisfies IS for some k induce IS at the level k + 1? The answer is negative. Even for low values of k, a Markov model based on a lower statistic cannot generate the higher statistic [8]. This issue has been discussed in [10] where the difference between the two has been termed “residual symmetry”.

**Table 4:** Evaluation of E[|Z|], E[X], fraction of unrealized inverse pairs, and chromosomal length

	k	HG38	HG38M	HG18	HG18M	Mouse	MouseM	C eleg	Cerevisiae	Ecoli
E[|Z|]	1	4.154	3.943	4.560	5.406	6.928	11.001	2.814	2.057	1.273
	2	2.581	2.316	3.260	3.417	3.695	5.652	1.548	1.682	1.479
	3	1.707	1.769	1.983	2.152	2.780	3.904	1.589	1.434	1.318
	4	1.446	1.392	1.339	1.492	1.809	2.342	1.397	1.000	1.012
	5	1.202	1.186	1.069	1.133	1.262	1.490	1.216	0.867	0.921
	6	1.057	1.001	0.930	0.943	0.990	1.070	1.075	0.791	0.852
	7	0.984	0.935	0.894	0.884	0.892	0.902	0.980	0.780	0.837
	8	0.929	0.883	0.867	0.845	0.843	0.839	0.893	0.787	0.815
	9	0.881	0.855	0.843	0.828	0.823	0.819	0.851	0.841	0.811
	10	0.844	0.831	0.825	0.816	0.815	0.813	0.824	0.902	0.815
	11	0.825	0.821	0.816	0.814	0.813	0.814	0.835	0.940	0.856
	12	0.824	0.829	0.821	0.826	0.822	0.828	0.881	0.956	0.916
E[X]	1	0.00038	0.00050	0.00041	0.00067	0.00068	0.00152	0.00099	0.00672	0.00083
	2	0.00046	0.00058	0.00058	0.00083	0.00070	0.00150	0.00111	0.01021	0.00196
	3	0.00067	0.00095	0.00077	0.00106	0.00121	0.00218	0.00260	0.01752	0.00350
	4	0.00134	0.00179	0.00115	0.00170	0.00165	0.00283	0.00474	0.02527	0.00554
	5	0.00247	0.00329	0.00206	0.00284	0.00260	0.00397	0.00839	0.04547	0.01067
	6	0.00470	0.00593	0.00402	0.00537	0.00461	0.00636	0.01535	**0.08576**	0.02075
	7	0.00942	0.01205	0.00852	0.01123	0.00941	0.01222	0.02905	0.18223	0.04362
	8	0.01918	0.02472	0.01809	0.02355	0.01954	0.02505	0.05593	0.38663	**0.08975**
	9	0.03951	0.05169	0.03850	0.04998	0.04226	0.05334	**0.11437**	0.64905	0.18551
	10	**0.08380**	**0.10979**	**0.08334**	**0.10736**	**0.09343**	**0.11601**	0.24551	0.82906	0.36850
	11	0.17518	0.22274	0.17538	0.21909	0.19196	0.23044	0.47655	0.91443	0.61571
	12	0.31969	0.38249	0.32051	0.37838	0.33829	0.38843	0.68957	0.94564	0.81471
Fraction of null pairs	7								0.00110	
	8								0.04863	0.00079
	9		0.00001		0.00001		0.00002		0.48397	0.00954
	10	0.00042	0.00130	0.00042	0.00127	0.00101	0.00217	0.00552	2.39166	0.06538
	11	0.01460	0.02590	0.01471	0.02515	0.02289	0.03312	0.14178	9.83436	0.30279
	12	0.09259	0.14336	0.09292	0.13934	0.11537	0.15551	0.85693	39.18	0.66052
length		2.3E + 08	1.1E + 08	2.2E + 08	1.2E + 08	1.9E + 08	1.1E + 08	1.5E + 07	230218	4639664

Displayed results are for chr1 of HG38, HG18, mouse, C elegans, and S cerevisiae, and for the full bacterial chromosome of E coli. M refers to masked chromomes. Centromere regions were removed from the HG 38 data. Highlighted results are the ones determining the k-limit, KL, of the different chromosomes

The simplest random model is that of a uniform distribution, which is generated on the basis of the second Chargaff rule (i.e. #A = #T and different from #C = #G). Such a distribution will trivially account for low E_k_ [X] values for inverse pairs at large values of k, limited by the length of the model chromosome. However it will also give rise to very low values for reverse pairs at a similar range of k, because any comparison of k-mers with one of their permutations will lead to similar E_k_[X]. In other words, this random independent (but not IID) model satisfies additional symmetries that are not observed in genomic data. Therefore it is not a realistic model of inversion symmetry.

A plausible explanation of the observed IS can be based on the fact that genomes evolve through rearrangement processes. By comparing synteny blocks in human and mouse, Pevzner and Tesler [17] have argued that rearrangements occur on many scales in the genome, and intra-chromosomal rearrangements are more frequent than inter-chromosomal ones. Rearrangements may be viewed as inversions of sections between two breakpoints on the chromosome, and they may even follow one another in a nested fashion. Their study [17] demonstrated that human and mouse chr X share 281 synteny blocks of size >1 Mb, and at least 245 rearrangements occurred since the divergence of the two species.

Building on this intuition, derived from comparative genomics, it seems reasonable to assume that a series of such rearrangements on different scales may lead to IS. This mechanism has already been suggested by [6], and has been studied by [18] and by [11]. We have tested it on a simple model, starting from the human mitochondrial chromosome, which does not satisfy the second Chargaff rule. Since the mitochondrial chromosome is only 16Kbp long, we first construct out of it an enlarged model chromosome with length L = 100Mbp, by concatenating random selections of subsequences of chr M. We then apply to it rearrangements at various scales. We found that 5000 rearrangements at scales of 100 K have led to good IS effects, but best results were obtained for 50,000 rearrangements, whose breakpoints were randomly chosen, and their section lengths befit a uniform random distribution of length < 10 K. These results exhibit a high degree of IS, as displayed in Additional file 7.

### Inversion symmetry: validation and deviations

Figure 4 provides an experimental validation of our IS-Poisson model, in so far that it predicts correctly the behavior of KL as function of ln(L).

To look further into it, we present in Table 4 a comparative analysis of chr1 of different species, in both its unmasked and masked formats, as well as the analysis of the E coli genome. Shown are E[|Z|] values, E[X] values, the fraction of unrealized pairs (where both N(S) = N(S^inv^) = 0), and the relevant length of the studied chromosomes. Highlighted are all E[X] values which are closest to 0.1 (defining the relevant KL) for the different chromosomes. By comparing with the upper part of the table one realizes that for k = KL, E[|Z|] is indeed close to 0.8, hence the success of the KL formula. By comparing with the 3^rd^ part of the table, we see that for these values of k, only for a very small fraction of all possible k-mers, both S or S^inv^ are not realized on the studied chromosomes.

Our criterion for approximate IS, E_k_[X] ≤ 0.1, was introduced as an intuitive but somewhat arbitrary decision. From Table 4 we learn that this is where the Z distribution approximates very well the data. For larger k we observe that some of the k-mers do not appear, and their fraction increases rapidly with k. Hence our criterion selects also the range where almost all k-mer strings are being realized. This serves as a posterior justification of our IS criterion.

Table 4 carries also the message that, for small k, a strict validity of IS cannot be guaranteed. This may be interpreted by stating that the breaking of IS is small, but it is statistically significant. In particular, testing the 2^nd^ Chargaff rule, one finds a systematic deviation from N(T) = N(A) and N(G) = N(C) for all human chromosomes, as displayed in Table 5. For most human chromosomes, we find an excess of T over A and of G over C on the positive strand. Only chr 8 and chr 22 display opposite trends. As shown, these results are statistically significant, when compared with an assumption that the counts of complement nucleotides are derived from the same Poisson distribution. Moreover, the same is true for both bare and masked versions of the chromosome. The difference between the bare and masked regions of the chromosome defined the low-complexity chromosomal regions. The asymmetry seems to be quite significant in all three regions (bare,masked,low complexity) as can be seen in Additional files 8 and 9.

**Table 5:** Violations of the 2^nd^ Chargaff rule on HG38. Columns contain the values of #T/#A, #G/#C on different chromosomes, as well as their Y and Z values. The latter reflect the significance of the inequality

	T/A	G/C	Y(T,A)	Y(G,C)	Z(T,A)	Z(G,C)
chr1	1.002593	1.001175	0.001295	0.000587	15	5.76
chr2	1.00274	1.002747	0.001368	0.001372	16.41	13.49
chr3	1.002416	1.002824	0.001207	0.00141	13.19	12.5
chr4	1.001062	1.002595	0.000531	0.001296	5.75	11.04
chr5	1.004679	1.004144	0.002334	0.002068	24.44	17.5
chr6	1.000537	1.001981	0.000268	0.000989	2.72	8.12
chr7	1.003332	1.001884	0.001663	0.000941	16.15	7.57
chr8	0.999241	1.002536	−0.00038	0.001266	−3.53	9.65
chr9	1.001327	1.002823	0.000663	0.001409	5.61	9.99
chr10	1.0039	1.002911	0.001946	0.001454	17.18	10.82
chr11	1.001915	1.002815	0.000956	0.001405	8.48	10.51
chr12	1.003102	1.003317	0.001548	0.001656	13.75	12.2
chr13	1.003831	1.005012	0.001912	0.002499	14.83	15.36
chr14	1.008943	1.007342	0.004451	0.003658	32.58	22.24
chr15	1.001842	1.00411	0.00092	0.002051	6.44	12.23
chr16	1.009601	1.007001	0.004778	0.003488	32.17	21.07
chr17	1.002905	1.006812	0.00145	0.003395	9.77	20.81
chr18	1.005494	1.016917	0.00274	0.008388	19.03	47.34
chr19	1.009276	1.007636	0.004617	0.003803	25.46	20.13
chr20	1.011147	1.012815	0.005542	0.006367	33.22	33.7
chr21	1.003017	1.005026	0.001506	0.002507	7.33	10.15
chr22	0.998893	1.009337	−0.00055	0.004647	−2.52	19.94
chrX	1.003463	1.005699	0.001728	0.002842	16.73	22.23
chrY	1.008873	1.000209	0.004417	0.000105	17.58	0.34

All Z values are very significant, but for Z(G,C) on chrY which corresponds to a p-value of 0.367. All other have inequality *p*-values < 0.01. On all chromosomes we observe #G > #C on the positive strand. Same is true for #T > #A, but for chr8 and chr22, where #T < #A, which is also a significant observation (|Z| > 2.575 corresponds to an inequality *p*-value < 0.005)

It is well-known that there exist local violations of the 2^nd^Chargaff rule; in particular, there exists an excess of #G over #C and #T over #A on the coding strand within most genes. Green et al. [19] have argued that mutational asymmetry has acted over long periods of time to produce such a compositional asymmetry, and discontinuities of such asymmetries are associated with loci of replication origin. These questions have also been studied by Huvet et al. [20]. Could it be that the asymmetry that we have encountered is somehow connected to these findings? Since the gene coding strand may be either the plus (P) or the minus (M) strand of the conventional genomic notation, this may seem to be unrelated, assuming there is equal probability for genes to occur on each strand.

The convention which is being used in the UCSC genome browser is that the “plus” strand refers to the linear 5’ to 3’ order of encountering the p-arm before the centromere, which is followed by the q-arm of the chromosome. This convention is consistent with NCBI “top” assignment. Counting protein coding genes and RNA genes on human chromosomes as recorded by GeneCards (http://www.genecards.org) we are led to the conclusion that they display a clear excess of genes on the plus (P) strands. The results are displayed in Table 6. Their relation to preferences of #T > #A and #G > #C on the P strand looks statistically significant. Clearly genes occupy only a small fraction of human chromosomes but they could still be the cause for the very small deviation from the Chargaff rule. It may also be that some other mechanism leads to a built-in excess on the chromosomes, and the latter affects the preference of gene allocations within the two strands. A notable exception to the observed general trend is chr 11.

**Table 6:** Gene occurrences on the plus (#P) and minus (#M) strands of HG38 display abundance of the former

chr	P	M	Y(P,M)	Z(P,M)	p values	Z(T,A)	Z(G,C)	corr
1	4488	4291	0.022	2.103	0.018	15.00	5.76	v
2	4106	3367	0.099	8.549	0	16.41	13.49	v
3	2938	2516	0.077	5.714	5.65E-09	13.19	12.50	v
4	2542	1792	0.173	11.392	0	5.75	11.04	v
5	2777	2186	0.119	8.389	0	24.44	17.50	v
6	4840	3563	0.152	13.931	0	2.72	8.12	v
7	3024	2402	0.115	8.444	0	16.15	7.57	v
8	2135	2032	0.025	1.596	**0.055**	*−3.53*	9.65	
9	3032	2180	0.163	11.802	0	5.61	9.99	v
10	2532	2156	0.080	5.492	2.01E-08	17.18	10.82	v
11	2879	4047	−0.169	*−14.035*	0	8.48	10.51	x
12	3003	2771	0.040	3.053	0.0011	13.75	12.20	x
13	1261	1227	0.014	0.682	**0.25**	14.83	15.36	
14	2092	1906	0.047	2.942	0.0016	32.58	22.24	v
15	4226	3547	0.087	7.702	6.77E-15	6.44	12.23	v
16	2529	1875	0.149	9.855	0	32.17	21.07	v
17	3582	2902	0.105	8.445	0	9.77	20.81	v
18	1182	1490	−0.115	*−5.958*	1.26E-09	19.03	47.34	x
19	3287	3036	0.040	3.157	0.00079	25.46	20.13	v
20	1258	1193	0.027	1.313	**0.09500**	33.22	33.70	
21	670	779	−0.075	*−2.863*	0.00212	7.33	10.15	x
22	1429	1793	−0.113	*−6.413*	7.28E-11	*−2.52*	19.94	?
X	1927	1572	0.101	6.001	9.87E-10	16.73	22.23	v
Y	491	184	0.455	11.816	0.00E + 00	17.58	**0.34**	
				*P < M*	***p*** **> 0.05**	*T < A*	***p*** **> 0.05**	

Three of the results are insignificant (highlighted ***p*** **> 0.05**, q > 0.044 using FDR corrections). Four chromosomes have opposite preferences, set in italics for *P < M* and *T < A*. For all significant results we find 16 chromosomes displaying both *P* > M, T > A, and G > C. Chr 22 has both *P* < M and T < A. Last column indicates significant correlations of T-A and G-C with gene counts (positive by v and negative by x)

There are two different issues which are noteworthy in Table 6. One is the correlation of the preference of #T > #A and #G > #C with the positive labeling of the strand. The other is the correlation of #T > #A and #G > #C with the preference for gene counts. Whereas the first may be coincidental (although it could be related to the labelling convention whose sources we were unable to trace), we believe that the second can be meaningful.

Next we looked for the violation of the 2^nd^ Chargaff rule on mouse and yeast, with the purpose of characterizing the asymmetries and looking for correlations with gene occurrences. The gene counts were obtained from MGI (MRK_list2 in ftp://ftp.informatics.jax.org/pub/reports/index.html) for mouse, and from SGD snapshot (http://www.yeastgenome.org/genomesnapshot) for yeast. While asymmetries of nucleotide occurrences are evident and significant in both, gene data are quite smaller than in human and no conclusive correlations can be deduced. The analyses are listed in Additional files 10 and 11. Finally we test the 2^nd^ Chargaff rule on C elegans and E coli in Additional file 12. While the former shows some significant inconsistencies, the latter is completely consistent. This behavior correlates well with the trends already noted in the first raw of Table 4, indicating large values of E[|Z|] for k = 1.

## Discussion

Inversion symmetry may be stated as the equality N(S) = N(S^inv^) where N are counts and S is some arbitrary string existing on a chromosome. Conventionally one studies such equalities over the space of all S which are k-mers of some given length k. In addition to this equality one requires that, if S^inv^ is replaced by other permutations over the space of all k-mers, analog rules will not hold.

After reinvestigating these questions on various genomic data, with special attention devoted to human data, we turned to a rigorous statistical study. For this purpose we defined the normalized differences Y = (N(S)-N(S^inv^))/(N(S) + N(S^inv^)) and X = |Y|. If the equality *N*(S) = *N*(S^inv^) holds for stochastic variables *N*, we expect the variable *Y* to have approximately Gaussian behavior. If, moreover, *N* is a Poisson distribution, then Z = (N(S)-N(S^inv^))/(N(S) + N(S^inv^))^½^ should have approximately a standard normal distribution. The stochastic variable *Z* is the appropriate one to be used for a z-test, characterizing the significance of IS values displayed by Y or X, under the IP-Poisson model.

In order to characterize approximate IS we have employed E_k_[X] ≤ 0.1 as a convenient measure. We saw in Table 4 that it captures the region for which significant results are obtained, and almost all k-mers appear on the chromosome. Defining the k-limit KL as the k-value for which E_k_[X] turns out to be closest to 0.1, we uncover a logarithmic increase of KL with chromosomal length. It turns out that this behavior is accounted for by our IS-Poisson model.

Our original definition of IS regarded it as an approximate symmetry. As such it was seen to be valid for all ranges of k up to KL. With the advent of the IP-Poisson model, we may investigate to what extent it can serve as an exact symmetry. It turns out that, for very low k, E_k_[X] though extremely low, is significantly different from 0. In other words, the confidence intervals derived from IS-Poisson, exclude a peak at Z = 0. This has lead us to investigate the violation of the 2^nd^ Chargaff rule, i.e. deviations from the relations N(T) = N(A) and N(G) = N(C). We find that deviations are very significant in human and in mouse, and quite significant on chromosomes of other eukaryotes. Moreover, in human we observe that, for most chromosomes, N(T) > N(A) and N(G) > N(C), i.e. these excesses are observed to occur on chromosomal plus (P) strands. Investigating the occurrences of genes on both strands, we find a similar excess with significant slight preference for the P strand. These results, for nucleotide excess and gene excess, are displayed in Table 6, and are seen to hold for a large majority of chromosomes.^1^ Still, there exist also some counter-examples. Could it be that the known asymmetries of complement nucleotides on gene coding strands are related to the observed correlation of the two effects in the human genome? This remains an interesting question for future studies.

## Conclusions

Inversion symmetry is valid for almost all chromosomes, even after filtering out their low-complexity regions. We have defined an empirical criterion of IS, and a corresponding k-limit (KL), which is the highest k for which all k-mer distributions abide by the symmetry. Analyzing the IS behavior using rigorous statistical methods, and comparing empirical results with our IS-Poisson model, we account for the universal increase of KL with respect to the chromosomal length.

For low k we find minute, yet significant, deviations from strict IS. This includes excess of counts of nucleotides T *vs* A and G *vs* C on positive strands of human chromosomes. We point out that this finding correlates with a significant (but small) excess of genes on the same positive strands.

## Methods

For a string S of length k, and its symmetry-related S*, we introduce the stochastic variables *N*(S) and *N*(S*), and through them the following variables *X*, *Y* and *Z* (using an italicized notation):

$$ \begin{array}{l}X\left(\mathrm{S},{\mathrm{S}}^{*}\right)=\left|N\left(\mathrm{S}\right)-N\left({\mathrm{S}}^{*}\right)\right|/\left(N\left(\mathrm{S}\right)+N\left({\mathrm{S}}^{*}\right)\right),Y\left(\mathrm{S},{\mathrm{S}}^{*}\right)=\left(N\left(\mathrm{S}\right)-N\left({\mathrm{S}}^{*}\right)\right)/\left(N\left(\mathrm{S}\right)+N\left({\mathrm{S}}^{*}\right)\right),\ \mathrm{and}\\ {}Z\left(\mathrm{S},{\mathrm{S}}^{*}\right)=\left(N\left(\mathrm{S}\right)-N\left({\mathrm{S}}^{*}\right)\right)/{\left(N\left(\mathrm{S}\right)+N\left({\mathrm{S}}^{*}\right)\right)}^{\raisebox{1ex}{$1$}\!\left/ \!\raisebox{-1ex}{$2$}\right.}.\end{array} $$

All three variables can be evaluated for each specific k-mer, hence they carry implicit indices of the space of 4^k^ k-mers. The empirical values of the different variables will be denoted by N, X, Y and Z. Measures similar to Y appear in the literature for k = 1 and k = 2 and are known as skews.

The symmetry-relation means that N(S) and N(S*) are drawn from the same distribution, *N*(S) = *N*(S*). Furthermore, it is reasonable to assume that k-mer appearances on a long chromosome resemble a Poisson process. This has been verified by us by investigating counts for all non-overlapping windows of some size L (e.g. L = 100 K on human chr 1). If the expectation of the Poisson is large enough (a typical quoted number is 30), we can safely assume by the Central Limit Theorem that the distribution of the statistic Z we define is well approximated by a standard normal distribution.

1. The semi-Gaussian distribution of inverse-pair differences.
  Let us consider a pair of k-mers, with counts N(S) and N(S*) respectively, for which we evaluate the ratios Y = (N(S) - N(S*))/(N(S) + N(S*)) and X = |Y|. Moreover, we assume that these counts are due to two random variables drawn from the same distribution, thus having the same average, E[N(S)-N(S*)] = 0, and follow a Gaussian distribution G = exp(−Y^2^/2 σ_G_^2^)/ σ_G_(2π)^½^. The counts of the distribution of X = |Y| will then follow a semi-Gaussian distribution P = 2 exp(−*X*^2^/2 σ_G_^2^)/ σ_G_(2π)^½^, defined for positive X only. The mean and variance of this semi-Gaussian are E[X] = σ_G_ (2/π) ^½^ ≈ 0.798σ_G_ and V[X] = σ_G_^2^ (1-2/π). Hence σ_X_ = 0.603σ_G_ = 0.755E_X_.
  Empirical verification of inverse pair distributions can be carried out by choosing counts for all non-overlapping windows of some size L (e.g. L = 100 K on human chr 1). Testing the X and Y distributions for inverse pairs of k = 8 we find the above description to be valid.
2. Poisson distributions of counts.
  Let us now assume that the counts N(S) observed on a chromosome, are realizations of stochastic variables which follow Poisson distributions, each with its own mean = variance. In the IS limit the distribution of the inverse N(S^inv^) coincides with that of N(S). Their difference should have a mean of 0, and variance which is the sum of the variances. Thus, for each inverse pair of k-mers, we expect Y = (N(S) - N(S^inv^))/(N(S) + N(S^inv^)) to become approximately Gaussian with mean 0 and standard-deviation σ_G_ =1/(N(S) + N(S^inv^))^½^. Alternatively we can state that Z = (N(S) - N(S^inv^))/(N(S) + N(S^inv^))^½^ should approximately follow a standard normal distribution with mean = 0 and variance = 1. It follows then, from the previous paragraph, that in this regime we should obtain, after averaging over all k-mers, the results E_k_(|Z|) = 0.8 and σ_k_(|Z|) = 0.6.
  Note that E_k_(|Z|) may also be viewed as E_k_(X/σ_X_), where σ_X_ = 1/(N(S) + N(S^inv^))^½^ for every particular k-mer under consideration. Tables 2 and 4 demonstrate that the experimental results for large k are close to the predicted theoretical expectation.
3. Monotonic increase of E_k_[X] as function of k in the IS limit.
  For perfect IS it is trivial to prove that E_k_[X] = 0 implies that this equality holds for lower k, i.e. E_k-1_[X] = 0. Here we study the case of approximate inversion symmetry, with the purpose of proving that small E_k_[X] < <1 implies even smaller E_k-1_[X]. For simplicity we assume that all 4^k^ k-mers are being realized on the chromosomal strings.
  Let {S_j_, j = 1…4^k^} be the set of all k-mers, and {S’_i_, i = 1…4^k-1^}be the set of all (k-1)-mers. Each (k-1)-mer can be extended to the right by one nucleotide, resulting in four k-mers. Let us refer to this extension of S’_i_ as the set S_jϵI_. Corresponding relations will hold for their inverse partners, extended by nucleotides to their left. It follows then that the counts of these sets can be related N(S’_i_) = ∑_jϵI_ N(S_j_). Hence
  $$ \begin{array}{l}{\mathrm{X}}_{\mathrm{k}\hbox{-} 1}\left(\mathrm{i}\right) = \left|\mathrm{N}\left(\mathrm{S}{'}_{\mathrm{i}}\right)\ \hbox{--}\ \mathrm{N}\left(\mathrm{S}{'_{\mathrm{i}}}^{\mathrm{i}\mathrm{nv}}\right)\right|/\left(\mathrm{N}\left(\mathrm{S}{'}_{\mathrm{i}}\right) + \mathrm{N}\left(\mathrm{S}{'_{\mathrm{i}}}^{\mathrm{i}\mathrm{nv}}\right)\right) = \left|\left({\displaystyle {\sum}_{\mathrm{j}\in \mathrm{I}}\mathrm{N}\left({\mathrm{S}}_{\mathrm{j}}\right)}\hbox{-}\ {\displaystyle {\sum}_{\mathrm{j}\in \mathrm{I}}\mathrm{N}\left({{\mathrm{S}}_{\mathrm{j}}}^{\mathrm{i}\mathrm{nv}}\right)}\right)\right|\ \\ {}/\ \left({\displaystyle {\sum}_{\mathrm{j}\in \mathrm{I}}\mathrm{N}\left({\mathrm{S}}_{\mathrm{j}}\right)} + {\displaystyle {\sum}_{\mathrm{j}\in \mathrm{I}}\mathrm{N}\left({{\mathrm{S}}_{\mathrm{j}}}^{\mathrm{i}\mathrm{nv}}\right)}\right).\end{array} $$
  Using the notation N(S_j_) – N(S_j_^inv^) = ∆N(S_j_), we note that the numerator on the right obeys |∑_jϵI_ ∆N(S_j_)| ≤ ∑_jϵI_ |∆N(S_j_)|. Because of varying signs this inequality may imply a strong decrease.
  We may now compare the expressions of E_k-1_[X] =4^-k+1^∑_i_X_k-1_(i) and E_k_[X] = 4^-k^∑_j_X_k-1_(j). Using the results of the previous paragraph we conclude that the numerators of X_k-1_(i) in E_k-1_[X] are smaller (or equal) than the numerators of X_k_(j), where jϵI, in E_k_[X]. Note however that the denominators of X_k-1_(i) and X_k_(j), where jϵI, are different. To the extent that all N(S_jϵI_) have similar values within the group jϵI when we approach the IS limit, this leads to X_k-1_(i) ≤ ∑_jϵI_ X_k_(j)/4, which implies that
  $$ {\mathrm{E}}_{\mathrm{k}\hbox{-} 1}\left[\mathrm{X}\right]\ \le {\mathrm{E}}_{\mathrm{k}}\left[\mathrm{X}\right]. $$
  In practice, for large k, we find in Tables 2 and 4 that E_k-1_ [X] ≈ E_k_[X]/2.
  It should be emphasized that the monotonic increase holds in the IS limit, i.e. when E_k_[X] < <1, but it is not a general property of k-mers on any chromosomal section. Synthetic counter examples can be constructed.
4. Comparison of E_k_[X] with the S1 measure.
  The measure S1, introduced by Baisnee Hampson and Baldi [10], comparing counts of all kmers with their inverses, is defined by
  $$ \mathrm{S}1=1{\textstyle \hbox{-} }{\displaystyle {\sum}_{\mathrm{i}}}\left|\mathrm{N}\left({\mathrm{S}}_{\mathrm{i}}\right){\textstyle \hbox{-}}\mathrm{N}\left({{\mathrm{S}}_{\mathrm{i}}}^{\mathrm{i}\mathrm{nv}}\right)\right|/{\displaystyle {\sum}_{\mathrm{i}}}\left(\mathrm{N}\left({\mathrm{S}}_{\mathrm{i}}\right)+\mathrm{N}\left({{\mathrm{S}}_{\mathrm{i}}}^{\mathrm{i}\mathrm{nv}}\right)\right). $$
  The denominator in this expression equals twice the length of the chromosome. The numerator may be regarded as an L1 distance between two sets of sequences.
  Note the difference from our measure E_k_[X], which may be written as
  $$ {\mathrm{E}}_{\mathrm{k}}\left[\mathrm{X}\right]={{\mathrm{M}}_{\mathrm{k}}}^{{\textstyle \hbox{-} }1}{\displaystyle {\sum}_{\mathrm{i}}}\left|\mathrm{N}\left({\mathrm{S}}_{\mathrm{i}}\right){\textstyle \hbox{-}}\mathrm{N}\left({{\mathrm{S}}_{\mathrm{i}}}^{\mathrm{i}\mathrm{nv}}\right)\right|/\left(\mathrm{N}\left({\mathrm{S}}_{\mathrm{i}}\right)+\mathrm{N}\left({{\mathrm{S}}_{\mathrm{i}}}^{\mathrm{i}\mathrm{nv}}\right)\right). $$
  E_k_[X] averages the relative difference of all k-mers on equal footing, whereas S1 sums all absolute differences.
  A comparison of the two different measures on human chr1 is presented in Table 7. We find that E_k_[X] is roughly twice (1-S1)_k_, and the latter is approximately equal to E_k-1_[X].

**Table 7:** Comparison of two measures of inversion symmetry on chr1 of HG18 and HG38

	HG18 chr1		HG38 chr1	
k	1-S1	E_k_[X]	1-S1	E_k_[X]
5	0.0016	0.0021	0.0072	0.009
6	0.0026	0.0040	0.010	0.013
7	0.0048	0.0085	0.014	0.017
8	0.0091	0.018	0.018	0.025
9	0.017	0.038	0.027	0.043
10	0.033	0.083	0.043	0.085

## Additional files

Additional file 1: The variation of k-limits (defined by largest k for which E_k_[X] ≈ 0.1) as function of chromosome length in HG38 both before and after masking has been applied. (DOCX 17 kb) Additional file 2: The variation of k-limits (defined by largest k for which E_k_[X] ≈ 0.1) as function of chromosome length in HG18 both before and after masking has been applied. (DOCX 17 kb) Additional file 3: Distribution of inverse pairs in a chromosomal section of length 10Mbp, drawn from chr1. Range of X < 0.1. (DOCX 100 kb) Additional file 4: Distribution of inverse pairs in a chromosomal section of length 1Mbp drawn from chr 1. Range of X < 0.3. Smoother distributions are obtained when k-mers containing CG dimers are excluded (not shown). (DOCX 165 kb) Additional file 5: Values of E_2_[X] for inverse pairs of k = 2, evaluated over non-overlapping windows (the ordinate specifies the serial number of the window) of length 5 K on chr1. Average value is 0.07. (DOCX 69 kb) Additional file 6: Jittery behavior of E_1_[X] for inverse pairs on non-overlapping windows of 1Kbp on chr 1 indicates semi-local violations of the second Chargaff rule. The ordinate specifies the serial number of the window. (DOCX 62 kb) Additional file 7: Histograms of inverse pairs for different k-mers evaluated on the model based on rearrangements applied to an artificial chromosome of length 1 M constructed out of the mitochondrial chromosome, as described in the text. Note that the distributions are confined within the very short range of X < 0.04. (DOCX 102 kb) Additional file 8: Z values for comparison of T and A counts on HG38. (DOCX 21 kb) Additional file 9: Z values for comparison of G and C counts on HG38. (DOCX 15 kb) Additional file 10: Mouse data: ratios of #T/#A, #G/#C, and numbers of genes on Plus and Minus strands, together with their Z values. Most gene ratios have insignificant Z values, i.e. they are consistent with equality. Most #T/#A and #G/#C display significant violation of strict Chargaff rule. ChrX is exceptional: here both Z values are small and Chargaff violation is insignificant. Gene data are derived from MRK_list2 in ftp://ftp.informatics.jax.org/pub/reports/index.html. (DOCX 18 kb) Additional file 11: Yeast data: ratios of #T/#A, #G/#C, and numbers of genes on Plus and Minus strands, together with their Z values. All gene ratios have low Z values, i.e. they are consistent with equality. Many #T/#A and #G/#C display significant violation of strict Chargaff rule. Gene data are derived from http://www.yeastgenome.org/genomesnapshot. (DOCX 17 kb) Additional file 12: Ratios of #T/#A and #G/#C and their Z values for C elegans and E coli. While for C elegans one observes some significant violations of the 2^nd^ Chargaff rule, the E coli data are completely consistent with this rule. (DOCX 15 kb)

## Footnotes

1 Skew analyses, i.e. non-vanishing Y(A,T) and Y(C,G), have been carried out before. One example is table 2 in [21]. The correlation with gene numbers on human chromosomes observed in Table 6 is new, to the best of our knowledge. Forsdyke et al. [22] have investigated the correlation of conventional positive (or “top”) strands with the difference of #A-#T in chromosomes of *C elegans* and *D melanogaster.* These organisms, which have low numbers of chromosomes, do not exhibit a clear preference for excess of either #A or #T.

## References

[1] Chargaff E (1950). Chemical specificity of nucleic acids and mechanism of their enzymatic degradation. Experientia.

[2] Chargaff E (1951). Structure and function of nucleic acids as cell constituents. Federal Proc.

[3] Crick F, Watson JD (1953). Molecular Structure of Nucleic Acids: A Structure for Deoxyribose Nucleic Acid. Nature.

[4] Rudner R, Karkas JD, Chargaff E (1968). Separation of B. subtilis DNA into complementary strands. III. Direct Analysis. Proc Natl Acad Sci U S A.

[5] Mitchell D, Bridge R (2006). A test of Chargaff’s second rule. Biochem Biophys Res Commun.

[6] Albrecht-Buehler G (2006). Asymptotically increasing compliance of genomes with Chargaff’s second parity rules through inversions and inverse transpositions. Proc Natl Acad Sci U S A.

[7] Prabhu VV (1993). Symmetry observations in long nucleotide sequences. *Nuc*. Acids Res.

[8] Baldi P, Brunak S. Bioinformatics, the machine learning approach. MIT Press. 2001

[9] Qi D, Cuticchia AJ (2001). Compositional symmetries in complete genomes. Bioinformatics.

[10] Baisnee P-F, Hampson S, Baldi P (2002). Why are reverseary DNA strands symmetric?. Bioinformatics.

[11] Kong S-G, Fan W-L, Chen H-D, Hsu Z-T, Zhou N, Zheng B, Lee H-C (2009). Inverse symmetry in complete genomes and whole-genome inverse duplication. PlosOne.

[12] Wang S, Tu J, Jia Z, Lu Z (2014). High order intra-strand partial symmetry increases with organismal complexity in animal evolution. Sci Rep.

[13] Afreixo V, Bastos CAC, Garcia SP, Rodrigues JMOS, Pinho AJ, Ferreira PJSG (2013). The breakdown of the word symmetry in the human genome. J Theor Biol.

[14] Powdel BR, Satapathy SS, Kumar A, Jha PK, Buragohan AK, Borah M, Ray SK (2009). A Study in Entire Chromosomes of Violations of the Intra-strand Parity of Complementary Nucleotides (Chargaff’s Second Parity Rule). DNA Res.

[15] Afreixo V, Rodrigues JMOS, Bastos CAC (2015). Analysis of single-strand exceptional word symmetry in the human genome: new measures. Biostatistics.

[16] Chor B, Horn D, Goldman N, Levy Y, Massingham T (2009). Genomic DNA k-mer spectra: models and modalities. Genome Biol.

[17] Pevzner P, Tesler G (2003). Genome rearrangements in Mammalian Evolution: Lessons from Human and Mouse Genomes. Genome Res.

[18] Okamura K, Wei J, Scherer SW (2007). Evolutionary implications of inversions that have caused intra-strand parity in DNA. BMC Genomics.

[19] Green P, Ewing B, Miller W, Thomas PJ (2003). NISC Comparative Sequencing Program & Green ED. Transcription-associated mutational asymmetry in mammalian evolution. Nat Gen.

[20] Huvet M, Nicolay S, Touchon M, Audit B, d’Aubenton-Carafa Y, Arneodo A, Thermes C (2007). Human gene organization driven by the coordination of replication and transcription. Gen Res.

[21] Mascher M, Schubert I, Scholz U, Friedel S (2013). Patterns of nucleotide asymmetries in plant and animal genomes. BioSystems.

[22] Forsdyke DR, Zhang C, Wei J-F. chromosomes as interdependent accounting units. J Biol Syst. 2010;18:1–16.

