# Supplementary material for: Inversion symmetry of DNA k-mer counts: validity and deviations
Source: BMC Genomics. 2016 Aug 31;17(1):696. doi: 10.1186/s12864-016-3012-8 (PMC5006273; doi:10.1186/s12864-016-3012-8)
Supplement: Additional file 1: — The variation of k-limits (defined by largest k for which Ek[X] ≈ 0.1) as function of chromosome length in HG38 both before and after masking has been applied. (DOCX 17 kb) [file 12864_2016_3012_MOESM1_ESM.docx]

The variation of k-limits (defined by largest k for which E_k_[x]≈0.1) as function of chromosome length in HG38 both before and after masking has been applied.
