# Supplementary figures and images for: Inversion symmetry of DNA k-mer counts: validity and deviations
Source: BMC Genomics. 2016 Aug 31;17(1):696. doi: 10.1186/s12864-016-3012-8 (PMC5006273; doi:10.1186/s12864-016-3012-8)

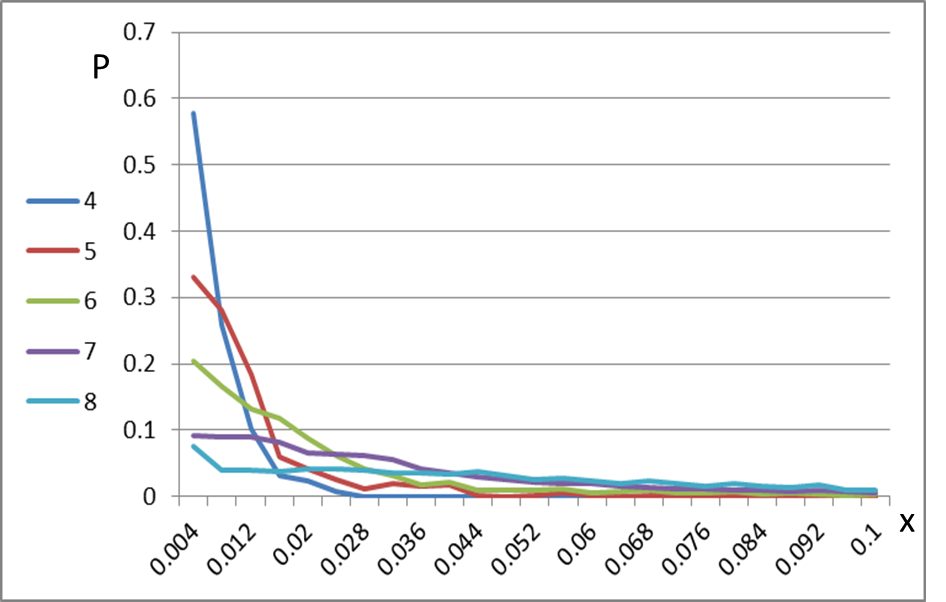


Distribution of inverse pairs in a chromosomal section of length 10Mbp, drawn from chr1. Range of X<0.1.

Supplement: Additional file 3: — Distribution of inverse pairs in a chromosomal section of length 10Mbp, drawn from chr1. Range of X < 0.1. (DOCX 100 kb) [file 12864_2016_3012_MOESM3_ESM.docx]
