# Supplementary material for: Inversion symmetry of DNA k-mer counts: validity and deviations
Source: BMC Genomics. 2016 Aug 31;17(1):696. doi: 10.1186/s12864-016-3012-8 (PMC5006273; doi:10.1186/s12864-016-3012-8)
Supplement: Additional file 4: — Distribution of inverse pairs in a chromosomal section of length 1Mbp drawn from chr 1. Range of X < 0.3. Smoother distributions are obtained when k-mers containing CG dimers are excluded (not shown). (DOCX 165 kb) [file 12864_2016_3012_MOESM4_ESM.docx]

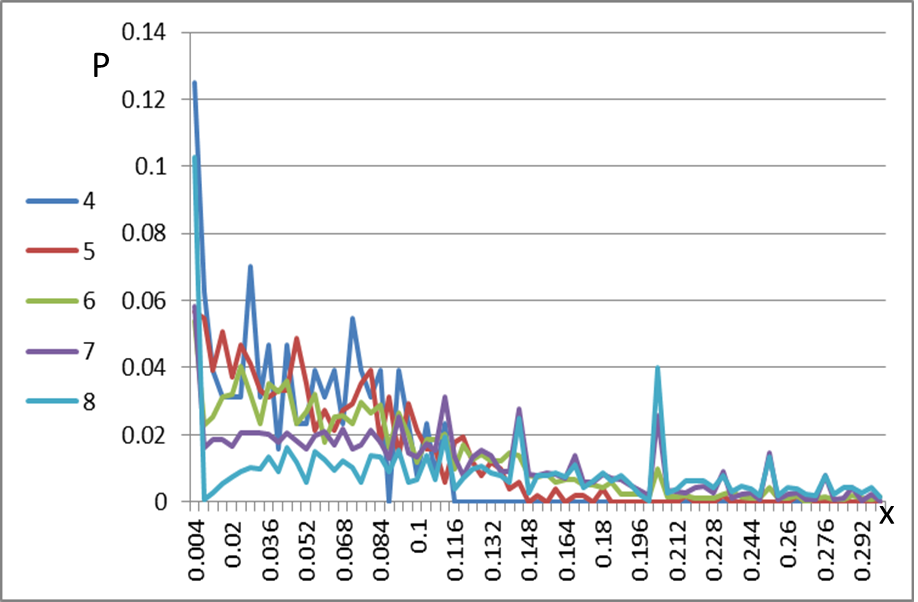


Distribution of inverse pairs in a chromosomal section of length 1Mbp drawn from chr 1. Range of X<0.3. Smoother distributions are obtained when k-mers containing CG dimers are excluded (not shown).
