# Supplementary material for: Inversion symmetry of DNA k-mer counts: validity and deviations
Source: BMC Genomics. 2016 Aug 31;17(1):696. doi: 10.1186/s12864-016-3012-8 (PMC5006273; doi:10.1186/s12864-016-3012-8)
Supplement: Additional file 5: — Values of E2[X] for inverse pairs of k = 2, evaluated over non-overlapping windows (the ordinate specifies the serial number of the window) of length 5 K on chr1. Average value is 0.07. (DOCX 69 kb) [file 12864_2016_3012_MOESM5_ESM.docx]

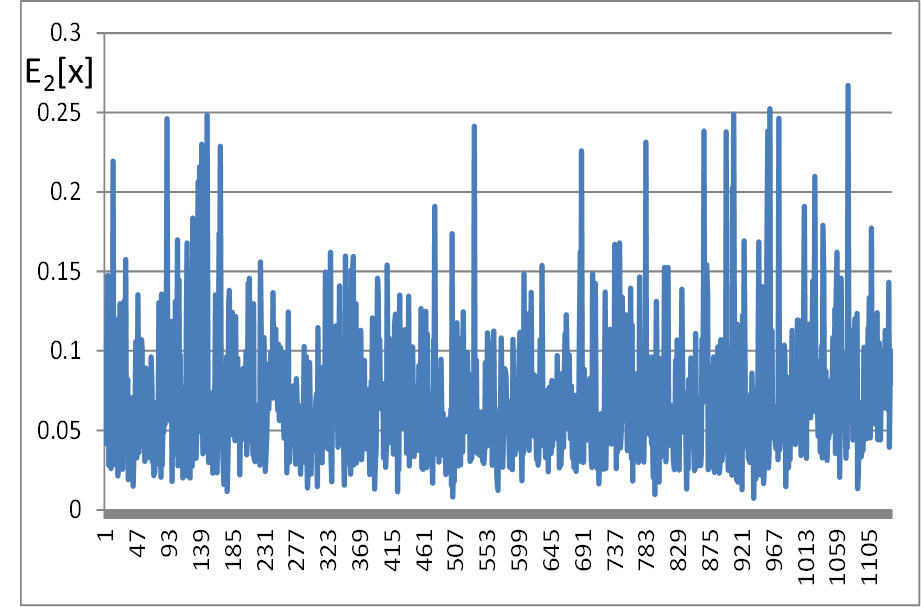


Values of E_2_[x] for inverse pairs of k=2, evaluated over non-overlapping windows (the ordinate specifies the serial number of the window) of length 5K on chr1. Average value is 0.07.
