# Supplementary material for: Inversion symmetry of DNA k-mer counts: validity and deviations
Source: BMC Genomics. 2016 Aug 31;17(1):696. doi: 10.1186/s12864-016-3012-8 (PMC5006273; doi:10.1186/s12864-016-3012-8)
Supplement: Additional file 6: — Jittery behavior of E1[X] for inverse pairs on non-overlapping windows of 1Kbp on chr 1 indicates semi-local violations of the second Chargaff rule. The ordinate specifies the serial number of the window. (DOCX 62 kb) [file 12864_2016_3012_MOESM6_ESM.docx]

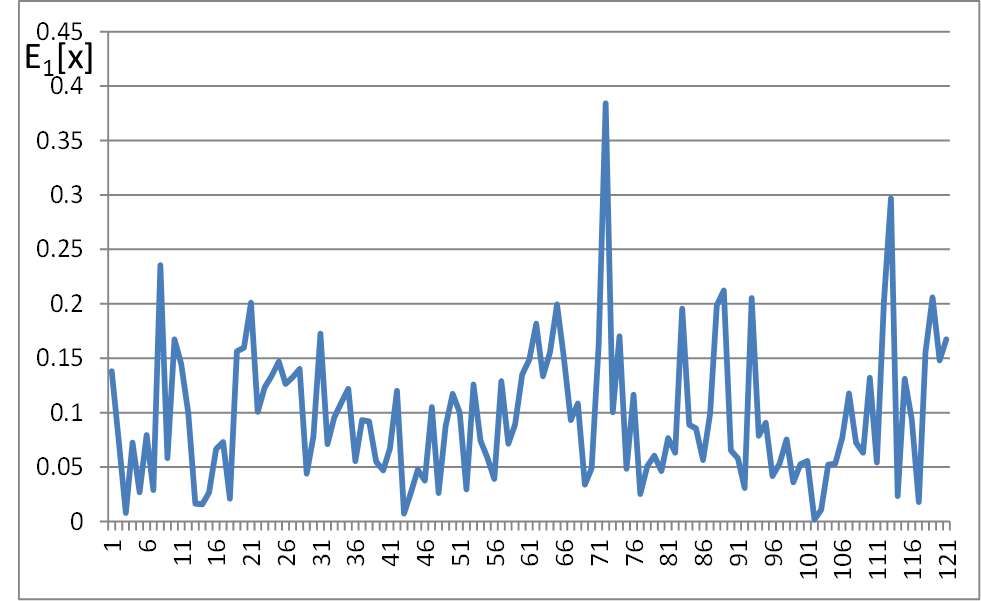


Jittery behavior of E_1_[x] for inverse pairs on non-overlapping windows of 1Kbp on chr 1 indicates semi-local violations of the 2^nd^ Chargaff rule. The ordinate specifies the serial number of the window.
