# Supplementary material for: Inversion symmetry of DNA k-mer counts: validity and deviations
Source: BMC Genomics. 2016 Aug 31;17(1):696. doi: 10.1186/s12864-016-3012-8 (PMC5006273; doi:10.1186/s12864-016-3012-8)
Supplement: Additional file 7: — Histograms of inverse pairs for different k-mers evaluated on the model based on rearrangements applied to an artificial chromosome of length 1 M constructed out of the mitochondrial chromosome, as described in the text. Note that the distributions are confined within the very short range of X < 0.04. (DOCX 102 kb) [file 12864_2016_3012_MOESM7_ESM.docx]

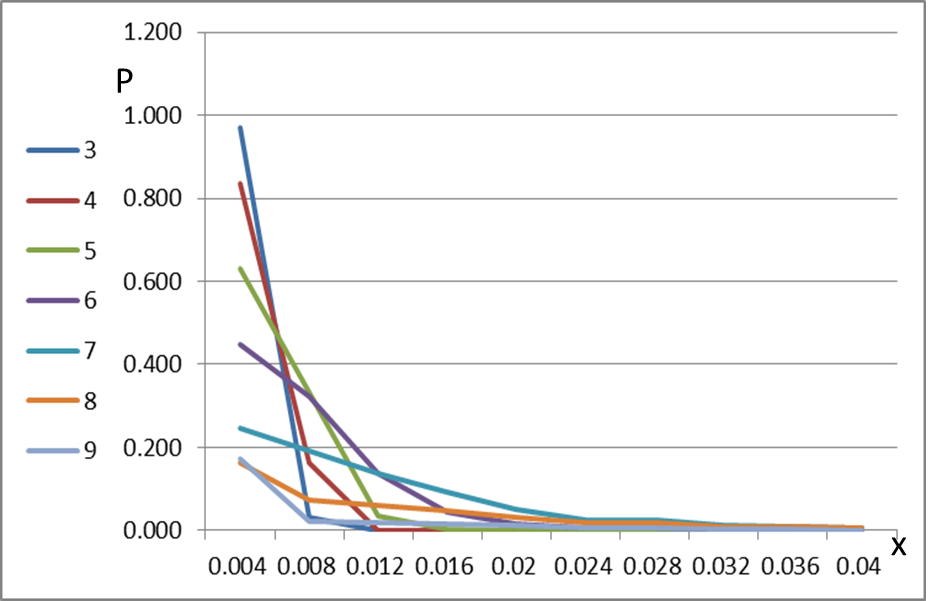


Histograms of inverse pairs for different k-mers evaluated on the model based on rearrangements applied to an artificial chromosome of length 1M constructed out of the mitochondrial chromosome, as described in the text. Note that the distributions are confined within the very short range of X<0.04.
