# Supplementary material for: Inversion symmetry of DNA k-mer counts: validity and deviations
Source: BMC Genomics. 2016 Aug 31;17(1):696. doi: 10.1186/s12864-016-3012-8 (PMC5006273; doi:10.1186/s12864-016-3012-8)
Supplement: Additional file 8: — Z values for comparison of T and A counts on HG38. (DOCX 21 kb) [file 12864_2016_3012_MOESM8_ESM.docx]

Z values for comparison of T and A counts on HG38.
