# Supplementary material for: Inversion symmetry of DNA k-mer counts: validity and deviations
Source: BMC Genomics. 2016 Aug 31;17(1):696. doi: 10.1186/s12864-016-3012-8 (PMC5006273; doi:10.1186/s12864-016-3012-8)
Supplement: Additional file 9: — Z values for comparison of G and C counts on HG38. (DOCX 15 kb) [file 12864_2016_3012_MOESM9_ESM.docx]

| HG38 | Zraw | Zmasked | Zlowcomp |
| --- | --- | --- | --- |
| chr1 | 5.759981 | 1.399834 | 6.640916 |
| chr2 | 10.81542 | 1.350985 | 13.93065 |
| chr3 | 10.50667 | 6.309937 | 8.502726 |
| chr4 | 12.19922 | 1.302003 | 15.36894 |
| chr5 | 15.3618 | -0.57071 | 22.00599 |
| chr6 | 22.24425 | 7.545172 | 23.5334 |
| chr7 | 12.23209 | -0.92503 | 18.00064 |
| chr8 | 21.0678 | 3.158153 | 26.36193 |
| chr9 | 20.80889 | -4.4234 | 33.41951 |
| chr10 | 47.34058 | 5.635347 | 60.2507 |
| chr11 | 20.13079 | 6.566452 | 20.907 |
| chr12 | 13.49205 | 4.998411 | 14.07325 |
| chr13 | 33.69652 | 8.294872 | 38.59204 |
| chr14 | 10.15446 | -1.58912 | 15.57935 |
| chr15 | 19.93651 | -0.92996 | 28.87054 |
| chr16 | 12.49672 | 2.412503 | 14.92741 |
| chr17 | 11.04032 | 2.564815 | 12.64057 |
| chr18 | 17.49799 | 3.295279 | 20.9569 |
| chr19 | 8.119586 | 3.68039 | 7.740639 |
| chr20 | 7.568284 | -3.69008 | 13.98354 |
| chr21 | 9.654848 | -3.29085 | 16.48972 |
| chr22 | 9.992853 | -0.89835 | 14.72111 |
| chrX | 22.23309 | 3.921352 | 25.02139 |
| chrY | 0.339836 | 6.432377 | -4.36116 |

Z values for comparison of G and C counts on HG38.
