# Supplementary material for: Inversion symmetry of DNA k-mer counts: validity and deviations
Source: BMC Genomics. 2016 Aug 31;17(1):696. doi: 10.1186/s12864-016-3012-8 (PMC5006273; doi:10.1186/s12864-016-3012-8)
Supplement: Additional file 10: — Mouse data: ratios of #T/#A, #G/#C, and numbers of genes on Plus and Minus strands, together with their Z values. Most gene ratios have insignificant Z values, i.e. they are consistent with equality. Most #T/#A and #G/#C display significant violation of strict Chargaff rule. ChrX is exceptional: here both Z values are small and Chargaff violation is insignificant. Gene data are derived from MRK_list2 in ftp://ftp.informatics.jax.org/pub/reports/index.html. (DOCX 18 kb) [file 12864_2016_3012_MOESM10_ESM.docx]

Mouse data: ratios of #T/#A, #G/#C, and numbers of genes on Plus and Minus strands, together with their Z values. Most gene rations have insignificant Z values, i.e. they are consistent with equality. Most #T/#A and #G/#C display significant violation of strict Chargaff rule. ChrX is exceptional: here both Z values are small and Chargaff violation is insignificant. Gene data are derived from MRK_list2 in <ftp://ftp.informatics.jax.org/pub/reports/index.html>.
