# Supplementary material for: Inversion symmetry of DNA k-mer counts: validity and deviations
Source: BMC Genomics. 2016 Aug 31;17(1):696. doi: 10.1186/s12864-016-3012-8 (PMC5006273; doi:10.1186/s12864-016-3012-8)
Supplement: Additional file 11: — Yeast data: ratios of #T/#A, #G/#C, and numbers of genes on Plus and Minus strands, together with their Z values. All gene ratios have low Z values, i.e. they are consistent with equality. Many #T/#A and #G/#C display significant violation of strict Chargaff rule. Gene data are derived from http://www.yeastgenome.org/genomesnapshot. (DOCX 17 kb) [file 12864_2016_3012_MOESM11_ESM.docx]

Yeast data: ratios of #T/#A, #G/#C, and numbers of genes on Plus and Minus strands, together with their Z values. All gene ratios have low Z values, i.e. they are consistent with equality. Many #T/#A and #G/#C display significant violation of strict Chargaff rule. Gene data are derived from <http://www.yeastgenome.org/genomesnapshot>.
