# Supplementary material for: Inversion symmetry of DNA k-mer counts: validity and deviations
Source: BMC Genomics. 2016 Aug 31;17(1):696. doi: 10.1186/s12864-016-3012-8 (PMC5006273; doi:10.1186/s12864-016-3012-8)
Supplement: Additional file 12: — Ratios of #T/#A and #G/#C and their Z values for C elegans and E coli. While for C elegans one observes some significant violations of the 2nd Chargaff rule, the E coli data are completely consistent with this rule. (DOCX 15 kb) [file 12864_2016_3012_MOESM12_ESM.docx]

Ratios of #T/#A and #G/#C and their Z values for C elegans and E coli. While for C elegans one observes some significant violations of the 2^nd^ Chargaff rule, the E coli data are completely consistent with this rule.
